# Data set for automatic detection of online misogynistic speech^☆^

**DOI:** 10.1016/j.dib.2019.104223

**Published:** 2019-08-22

**Authors:** Theo Lynn, Patricia Takako Endo, Pierangelo Rosati, Ivanovitch Silva, Guto Leoni Santos, Debbie Ging

**Affiliations:** Dublin City University (DCU), Irish Institute of Digital Business (dotLAB), Brazil

**Keywords:** Misogyny detection, Misogynistic speech, Hate speech, Online speech, Urban dictionary

## Abstract

The data set is composed of 2285 definitions posted on the Urban Dictionary platform from 1999 to May 2016. The data was classified as misogynistic and non-misogynistic by three independent researchers with domain knowledge. The data set is available in public repository in a table containing two columns: the text-based definition from Urban Dictionary and its respective classification (1 for misogynistic and 0 for non-misogynistic).

**Table:** Specifications Table

Subject area	*Computer science.*
More specific subject area	*Social media, hate speech, misogyny speech, misogynistic speech detection, language and linguistics.*
Type of data	*Text file (table format).*
How data was acquired	*Content classification was done based on online Urban Dictionary definitions and the data was gathered using Urban Dictionary API.*
Data format	*The data was retrieved from Urban Dictionary, classified and made available in a text file (table format).*
Experimental factors	*Data consist of classified Urban Dictionary definitions (misogynistic content or not).*
Experimental features	*Social media platforms are being heavily used by people to publish and express their thoughts, and to practice their freedom of expression; as consequence a proliferation of online harassment, including hate speech generally and misogynistic speech specifically, is also being experienced in such online platforms.*
Data source location	*Online at*https://data.mendeley.com/datasets/3jfwsdkryy/3
Data accessibility	*Data set is in public repository.*
Related research article	*A Comparison of Machine Learning Approaches for Detecting Misogynistic Speech in Urban Dictionary*[1]

**Table:** 

**Value of the data** • This data set can be used by other researchers to implement automatic mechanisms (through machine learning, for instance) to detect misogynistic speech based on Urban Dictionary terms; • This data set can also be used together with other data sets (e.g. Twitter and Facebook) in order to further develop hate speech analysis and other detection mechanisms using multimodal text source; • The amount of data available is good enough for training, validating and testing machine learning models, however further expansion of the data set would be extremely valuable.

## 1 Data

The data set available in this paper is composed of 2285 definitions gathered from Urban Dictionary [2]. After a classification process done by three independent researchers, 1034 definitions were classified as misogynistic and 1251 as non-misogynistic.

## 2 Experimental design, materials, and methods

All data were downloaded using Urban Dictionary API [3]. The original data set was composed of 2,606,521 definitions posted by 2,001,482 distinct users between the launch of the Urban Dictionary platform in 1999 and May 2016. After a filtering process (described next), the data set available in this paper was created and it is composed of 2285 definitions.

A list of 51 words typically associated with misogynistic content was created by one researcher with extensive domain knowledge. This bag of words was used to filter 951,978 potentially misogynistic definitions out from the initial data set. The rationale behind pre-filtering the data set is that potentially misogynistic words can be use in non-misogynistic sentences too (e.g. “ass fucking lesbians” as misogynistic; “misspelling of lesbian” as non-misogynistic).

A sample of 2285 definitions was extracted and manually classified by two independent researchers. Disagreements were resolved by a third one. Out of those 2285 definitions, 1034 were classified as misogynistic and 1251 as non-misogynistic.

### 2.1 Ethical considerations

The data set was anonymized before its publication. Warning: This data set contains material that many will find offensive.
