# Supplementary material for: Data set for automatic detection of online misogynistic speech
Source: Data Brief. 2019 Aug 22;26:104223. doi: 10.1016/j.dib.2019.104223 (PMC6726860; doi:10.1016/j.dib.2019.104223)
Supplement: Multimedia component 1 [file mmc1.pdf]

A Comparison of Machine Learning Approaches for Detecting Misogynistic Speech in Urban Dictionary

Theo Lynn
Irish Institute of Digital Business
Dublin City University
Dublin, Ireland
theo.lynn@dcu.ie

Patricia Takako Endo
Irish Institute of Digital Business
Dublin City University
Dublin, Ireland
patricia.endo@dcu.ie

Pierangelo Rosati
Irish Institute of Digital Business
Dublin City University
Dublin, Ireland
pierangelo.rosati@dcu.ie

Ivanovitch Silva
Digital Metropolis Institute
Federal University of Rio Grande do Norte
Natal, Brazil
ivan@imd.ufrn.br

Guto Leoni Santos
Federal University of Pernambuco
Recife, Brazil
guto.leoni@gprt.ufpe.br

Debbie Ging
Institute for Future Media and Journalism
Dublin City University
Dublin, Ireland
debbie.ging@dcu.ie

***Abstract*—Recent moves to consider misogyny as a hate crime have refocused efforts for owners of web properties to detect and remove misogynistic speech. This paper considers the use of deep learning techniques for detection of misogyny in Urban Dictionary, a crowdsourced online dictionary for slang words and phrases. We compare the performance of two deep learning techniques, Bi-LSTM and Bi-GRU, to detect misogynistic speech with the performance of more conventional machine learning techniques, logistic regression, Naive-Bayes classification, and Random Forest classification. We find that both deep learning techniques examined have greater accuracy in detecting misogyny in the Urban Dictionary than the other techniques examined.**

***Index Terms*—misogyny, hate speech, recurrent neural networks, deep learning, LSTM, machine learning, urban dictionary**

I. Introduction

The concept of hate speech emerged in the 1980s as a means to highlight how different legal systems deal with the tension between racist speech and protected speech [1]. While it is widely used, a common definition is difficult to identify. A review of definitions by Richardson-Self [2] suggests that hate speech is characteristically hostile, is targeted at a group of people with actual or imagined traits (race, religion, sexual orientation, disability, gender status, gender identity etc.), and seeks to achieve certain negative actions against these groups e.g. to silence, malign, humiliate, intimidate, incite violence, discriminate etc. Misogyny is typically defined as "[...] *a cultural attitude of hatred for females because they are female*" [3]. Manne [4] argues that misogyny is a property of social systems as a whole, particularly those that have evolved from a system of patriarchal oppression. As such, misogynistic behaviour may be evidenced in everyday behaviour, consciously and subconsciously, by both men and women, offline and online. The seriousness and implications of misogynistic behaviour is increasingly a public security issue. For example, in the UK, Nottinghamshire Police has recorded misogyny as a hate crime [5] and in September 2018, it was announced that the UK Law Commission would review whether misogynistic conduct should be treated as a hate crime [6].

Given the widespread use of the Internet and social media by all facets of society, it is unsurprising that misogynistic behaviour is evident online [7]. While dramatically increasing the public's ability to publish and give voice to their freedom of expression, it has also resulted in a proliferation of online harassment including hate speech generally and misogynistic speech specifically on social media [8]–[10]. Recent research by Amnesty International across eight countries, suggested that 23 per cent of the women surveyed had experienced online abuse or harassment at least once, and 41 per cent of these women had experienced online incidents that made them feel that their physical safety was threatened [11]. Similarly, one study has found evidence of that use of misogynistic language on social media is correlated with higher number of rapes per capita at the level of states [12]. Against this backdrop, it is not surprising that there is increase legal focus on policing and prosecuting online abuse but also that 79 per cent feel that online services have a responsibility to intervene when harassing behaviour occurs on their platforms [13].

As well as the social and legal drivers for detecting and intervening to curb such behaviour, there are economic reasons too. For most online communities and social networking sites, advertising is a cornerstone of their business models, and brands simply do not want to alienate their markets by being associated with anti-social behaviour. For instance, this was clearly illustrated in 2017 instance when several major brands, including AT&T, Walmart, Pepsi and others, pulled their advertising from YouTube after their adverts were found to be appearing next to videos promoting extremist views or hate speech [14].

Due to the scale of user populations on modern internet platforms,the volume of content generated and the legal, regulatory and reputational risk associated with objectional content,

online service providers are increasingly using a combination of flagging, human content moderation, and automated abuse detection systems to detect potentially abusive behaviour in a timely and cost-effective manner [15], [16]. The empirical context for this paper is Urban Dictionary, a crowdsourced online dictionary for slang words and phrases.[1] It is the 42nd most popular site in the US with over 80 million monthly users and 180 million monthly page views [17]. Dictionaries are powerful cultural and historical forces that provide meanings for verbal signs at a given time [18]. Due to its crowdsourced nature, Urban Dictionary is often used to capture and understand neologisms that may not be captured by traditional lexicography.

In this paper, we focus on the use of deep learning techniques for the automatic detection of misogynistic speech in Urban Dictionary terms in the English language. While there is a nascent but growing corpus of studies using machine learning for identifying hate speech, there are relatively few studies on automatic misogyny detection, particularly using deep learning techniques or Urban Dictionary as an empirical context. We present two deep learning models trained to identify misogynistic language based on bidirectional long short-term memory recurrent neural networks (Bi-LSTM) and bidirectional gated recurrent unit recurrent neural networks (Bi-GRU). We compare the performance of these deep learning networks to detect misogynistic speech with more conventional machine learning classifier techniques, namely Logistic Regression, Naive-Bayes, and Random Forest.

The remainder of the paper is organised as follows. Section II provides an overview of machine learning and then discusses recurrent neural networks, so-called deep learning. Section III presents the deep learning networks used in our study to detect misogynistic speech. This is followed by a discussion of results comparing deep learning techniques to more traditional machine learning techniques in Section IV. Section V discusses extant studies on the use of deep learning to detect misogynism in online content. The paper concludes with a summary of our study, challenges and suggested avenues for future research.

II. Background

Over the last two decades, the prevalence of machine learning has risen dramatically both in academic studies and industrial applications. It has been applied in a wide variety of use cases including financial services [19], healthcare [20], and manufacturing [21]. The interest in machine learning is evident in the proliferation of techniques that have been emerged and combined to address problems in these contexts including Support Vector Machine (SVM), Random Forest, Naive-Bayes classification, amongst others [19].

While there is increasing evidence regarding the suitability of machine learning to deal with large volumes of data quickly for improved decision making, there are classes of problems where conventional machine learning techniques may be sub-optimal such as processing raw time series data. Such data is widespread and increasing in volume in line with the emergence of the so-called, Internet of Everything [22]. In these scenarios, people, machines (incl. sensors), and processes generate massive volumes of data. Such raw time series data is problematic for conventional machine learning techniques because it assumes that all inputs are independent of each other. Consequently, an internal and abstract representation, often referred to as features, needs to be extracted manually based on domain knowledge resulting in greater cost, both in terms of time and effort [23]. New techniques, such as neural networks, have emerged to solve the need for handcrafted feature extraction from raw data however these also result in a trade off between accuracy and deliberation time and associated costs [24]. More recently, a new paradigm of neural networks has emerged, data-driven deep learning. Its use by both Google[2] and Facebook[3] to achieve faster decision times and greater accuracy than conventional machine learning, is attracting significant attention in academia and industry.

A. *Deep learning*

Deep learning is a sub-field of neural networks that uses non-linear connected modules, called neurons. Commonly, these neurons are arranged in sequential layers, and the output of one layer is used as input of the next layer (feedforward), as illustrated in Figure 1.

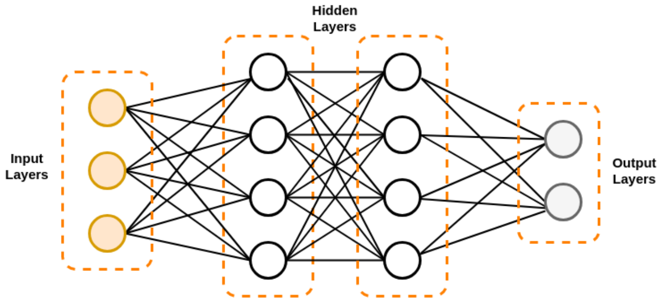

Fig. 1: Example of generic neural network (based on [23])

1) Recurrent Neural Network: Recurrent Neural Networks (RNN) is a variation of traditional feed-forward neural networks designed to deal with tasks that involve sequential inputs, such as text analysis, temporal series, and speech recognition [25] [26]. While traditional neural networks are limited to fixed-size sliding on the data, RNNs contain cycles in their units that maintain the output of previous time step i.e. to keep the history of all past elements of the training data. Thus, the previous time steps influence the output of the current time step [27].

Figure 2 illustrates an example of a recurrent unit. The current time step, x_t, takes into account the stored information of the previous time step (x_{t-1}), and its output is the input for the next time step, x_{t+1}. The same weight matrices (*U, W, and V*) are used at each time step. Therefore, RNNs can map

[1]https://www.urbandictionary.com

[2]https://deepmind.com

[3]https://https://research.facebook.com/ai/

the input sequence x_t into an output sequence o_t, where each o_t depends on all previous data (for $t' \leq t$).

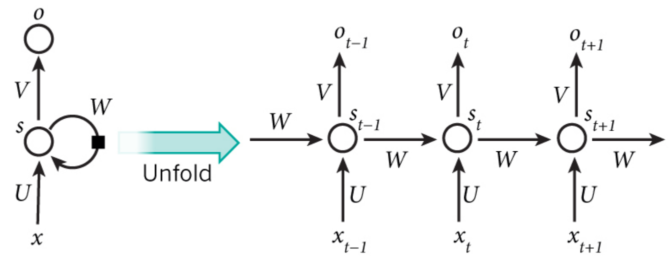

Fig. 2: Recurrent unit of RNN (adapted from [26])

Although RNNs are a powerful technique to deal with sequential data, the vanish gradient problem makes them hard to train using back propagation algorithms [28] [26]. This problem occurs during the training phase when the gradient propagated through the network in the training either decays or grows exponentially. Some variants of traditional RNN units such as Long Short-Term Memory (LSTM) and Gated Recurrent Unit (GRU) provide useful solutions to this challenge.

2) LSTM: An LSTM network is a variation of traditional RNN, where its memory cell (also called unit) acts as an accumulator of the state information [26] [29].

The operation of a memory cell is controlled by three gates: input gate, forget gate, and output gate. Whenever a new input arrives, the information is accumulated to the cell if the input gate activates. The information about the previous data can be forgotten when the forget gate activates. This process is made through a sigmoid function, which outputs between zero and one; where zero means completely keep, and one means completely forget.

Finally, the output gate controls the output of the cell according to the input data. Thus, using these gates to control the information flow through the memory cells, the gradient will be trapped within the cell, preventing the vanish problem [29]. In addition, LSTM networks can learn complex long-term temporal dynamics that traditional RNN networks are incapable of [30].

3) GRU: GRU is a variation of LSTM which also adopts a gate concept to modulate the information flow inside the units [31]. However, GRU networks have only two gates: a reset gate, which is the equivalent of a forget gate for an LSTM, and an update gate, which is the equivalent of an input gate for an LSTM. Unlike LSTM, GRU fully exposes its memory content at each time step while trying to balance existing and new memory content using leaky integration with its adaptive time constant controlled by the update gate. This update mechanism allows a GRU to capture long-term dependencies, as with LSTM networks [32].

4) Bidirectional RNNs: The traditional RNN architectures are able only to take into account the past information of each specific time step [33]. However, in some applications, it is necessary to evaluate the whole input sequence. For instance, in speech recognition, the correct interpretation of the current sound depends on the proceeding phonemes [34]. Similarly, when analysing text, the meaning of a word can be affected by the proceeding words providing which compose the context of the entire sentence.

Bidirectional RNNs (bi-RNNs) were created to overcome these types of problem. In a bi-RNN, the hidden layers are able to process the input sequence both forward and backward with two separate hidden layers. The backward and forward layer outputs are then combined to produce a new output that is forwarded to the next hidden layers [33]. In this context, the output of each time step depends on the inputs from both the past and the future, therefore the final output includes more nuanced clues related to the context of each specific entry. In this paper, we use two types of Bi-RNN: Bi-LSTM and Bi-GRU.

B. Testing and Validation

A common procedure in machine learning application is to test how well a model will perform on new cases. One way to achieve that is to split your data into two sets: a training set and a test set. A model is initially trained using the training set; then its performance is evaluated based the accuracy of its predictions on the test set. The difference between model predictions and actual instances (that the model did not read during the training) will determine its accuracy.

A typical problem with machine learning algorithms is over-fitting. This occurs when the error on the training set is low whereas the error on the test set is high. Theoretically, over-fitting can be solved using different approaches, such as regularization, dropout, hyperparameter tuning [35]. However, if the model is trained many times using different hyper-parameters or other techniques using the same test set, the generalization error will be compromised because the model will adapt for that set. One strategy to solve this problem is to have a second test set, which is typically referred to as "validation set". Here, several models are trained using a limited set of hyperparameters and the algorithm that performs best on the validation set is selected. Once the accuracy requirements are reached, the generalisation error can be evaluated under the test set. This technique is described in Figure 3.

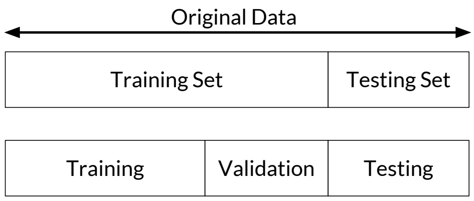

Fig. 3: A holdout approach to split original data into train, validation and test sets [36].

A drawback of the validation technique is that portion of the training data is wasted on validation. This can be particularly problematic when the training set is small. A

possible solution to this is using k-fold cross-validation [37]. In this approach, the whole training set is split into k folds (subsets). A combination of $k-1$ folds is used to train the model over k iterations while the remaining fold is used for validation purposes. An average score is used to measure the generalisation error. Figure 4 describes the cross-validation technique used in this study.

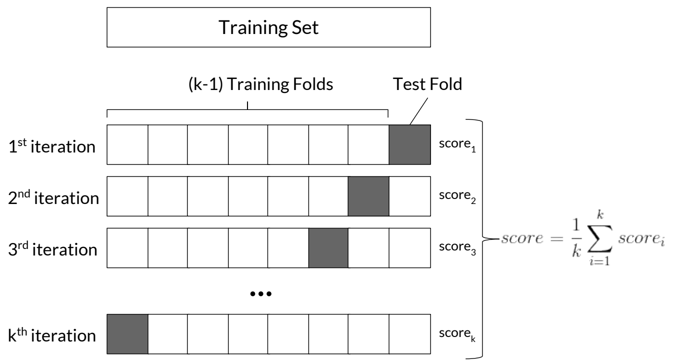

Fig. 4: A k-fold cross-validation approach [38].

III. Detecting hate speech

In this section, we present the three conventional machine learning classifiers and the two deep learning models used in this paper to detect misogynistic speech in Urban Dictionary. We particularly focus on the deep learning models as they are more novel than other more traditional machine learning techniques.

A. Conventional Machine Learning

In this paper, the three conventional machine learning algorithms for text classification implemented were Naive Bayes, Logistic Regression and Random Forest. Naive Bayes is a probabilistic classifier based on the Bayes' theorem, widely used for text classification purposes [39]. Naive Bayes classifiers are simple and easy to implement, access disk-resident data efficiently, and computational efficient, but they may show mediocre accuracy [40]. Logistic regression is a well-established statistics technique built upon a body of supporting theory [41]. Despite the fact that its predictive accuracy is often comparable with the one of more sophisticated techniques, traditional maximum likelihood estimation struggles in provide good performance for applications where the number of predictor variables exceeds the number of observations e.g. text classification [42]. Random Forest combines a forest of decision trees developed on randomly sampled subspaces of input vectors and then aggregates the resulting outputs by voting for the most popular class [43] [44]. Random forest algorithms typically achieve superior generalization on small training samples, but are computationally intensive [45]. All the algorithms were trained on 80 per cent of the sample and tested on the remaining 20 per cent.

First, a simple Naive Bayes classifier was created. Every definition is viewed as a collection of words [46] with the algorithm estimating the probability of each word occurring within a misogynistic or non-misogynistic definition.

Secondly, a Logistic Regression classifier was designed levering n-grams as proposed by [47]. The regression parameters were estimated using a generalized linear model and the number of iterations was determined in order to maximize the ratio between true and false positives. Thirdly, the Random Forest algorithms were configured. The Random Forest algorithms had 50 classification trees as it provided better performance than other configurations. Also this number of trees should be large enough to let the generalization error converge. A key parameter, to which Random Forests is somewhat sensitive, is the number of variables randomly sampled to split each node on [44]. To this end, we evaluate the performance of Random Forests with different numbers of random variables and selected the one with the best performance i.e. 4 variables.

B. Deep learning networks

We propose two bidirectional models with different types of recurrent layers: LSTM (bi-LSTM) and GRU (bi-GRU). But, we need to pre-process the data in order to fit it into our models.

Firstly, we convert each sequence into a vector of numbers in order to use it as input to the bidirectional models. For this, we apply the one-hot encode technique that encodes text into a list of integers [48]. Each sequence created may have a different size. To provide the models with inputs of the same length, we use padding to transform the sequences to the same length without impacting the semantics of the sequence [49].

Next, we define an embedding layer to create the dense vector of each sequence in order to capture the characteristics of the neighbours of a word. This type of layer is often used as the first layer in deep learning models [50]. The input size of the embedding layer represents the vocabulary size. We set it equal to the size of the largest definition present in our data set. While this is greater than needed, it reduces the probability of two words occurring where they are both associated to the same integer number. We define the dimension of the dense embedding as equalling 32. We use the same embedding layer configuration for both bi-LSTM and bi-GRU models.

Our bi-LSTM network is composed of three bidirectional layers containing 50 LSTM cells each as illustrated in Figure 5. In the cell configuration, we use the tangent hyperbolic as activation function and hard sigmoid as recurrent activation function. In order to reduce over-fitting, we add a dropout layer with the probability of 30% after each bidirectional layer. Afterwards, we added a fully connected layer with two cells, using sigmoid as the activation function. Each cell represents one of the expected outputs i.e. it is misogynistic or not. We use L2 regularization to reduce the over-fitting in this layer.

Our bi-GRU network is similar to the bi-LSTM. The main difference is that we use only two bidirectional GRU layers instead of three (Figure 6). We also have 50 GRU cells in each layer using tangent hyperbolic as the activation function, and hard sigmoid as the recurrent activation function. After each bidirectional layer, we added a dropout layer with 15%

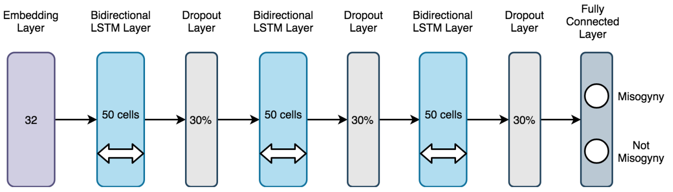

Fig. 5: Bidirectional LSTM network

of deactivation probability in order to reduce over-fitting. The fully connected layer is similar to the bi-LSTM network: two cells with each one using sigmoid as the activation function and L2 regularization to reduce the over-fitting.

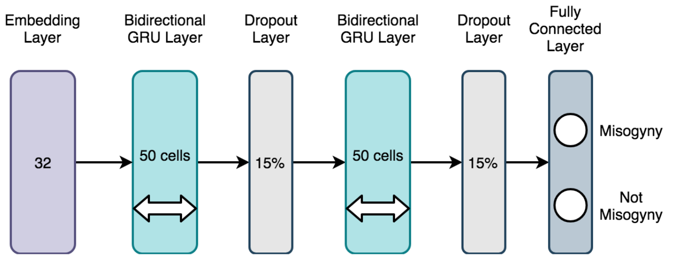

Fig. 6: Bidirectional GRU network

We used k-fold cross validation scheme (see sub-section II-B) with $k = 10$ to train our deep learning models. The training and the test set consist of 2,056 and 229 records respectively. We used Mean squared error as loss function, and the AMSGrad [51] - a variation of the Adam algorithm [52] - as optimizer. The Adam algorithm parameters were as follows: learning rate equals to 0.001; β_1 and β_2 equals to 0.9 and 0.999. During the k training processes, we used 50 epochs with batch size equalling to 64. All these parameters were configured empirically.

IV. Methodology

A. Dataset analysis

The original dataset included all the entries 2,606,521 definitions (1,552,312 terms) as posted by 2,001,482 distinct users between the launch of the platform in 1999 and May 2016. All the entries were downloaded using Urban Dictionary API[4].

A list of 51 words typically associated with misogynistic content was created by one of the researchers with extensive domain knowledge. This bag of words was used to filter 951,978 potentially misogynistic definitions out from the full data set. The rationale behind pre-filtering the data set is that potentially misogynistic words can be use in non-misogynistic sentences too (e.g. "ass fucking lesbians" as misogynistic; "mispelling of lesbian" as non-misogynistic). Therefore, by training the algorithms on a sample of potentially misogynistic content, they should be better able to discern between true and false positives.

For this purpose, a random sample of 2,285 definitions was extracted and manually coded by two independent coders. Disagreements were resolved by one of the authors with experience in the field. Out of those 2,285 definitions, 1,034 were classified as misogynistic and 1,251 as non-misogynistic.

B. Metrics

We used the three following metrics to compare the models: accuracy, sensitivity, and specificity.

Accuracy is "*the rate of correct classification*" [53] and it is calculated as:

$$acccuracy = \frac{TP + TN}{TP + FP + TN + FN} \quad (1)$$

where TP is true positive, TN is true negative, FP is false positive, and FN is false negative.

Sensitivity (or true positive rate) is the "*probability of a positive test result among those having the target condition*" [54]. The following equation is commonly used to calculate the sensitivity [55] [56] [53] [57]:

$$sensitivity = \frac{TP}{TP + FN} \quad (2)$$

Specificity (or true negative rate) is the "*probability of a negative test result among those without the target condition*" [54] and can be calculated use the following equation:

$$specificity = \frac{TN}{TN + FP} \quad (3)$$

C. Models comparison

Table I shows the results regarding the test phase. Naive Bayes achieved the worst result across all metrics (once it is the baseline reference), while Bi-GRU and Bi-LSTM provided the best results in terms of accuracy (93.10% and 90.12%, respectively) and sensitivity (92.08% and 87.53%, respectively), and Random Forest reached the best result in terms of specificity (96.00%).

As the data set adopted in this work is slightly imbalanced (it includes 1,034 positive instances (misogynistic definitions) and 1,251 negative instances (non-misogynistic definitions)),

[4]https://github.com/mattbierner/urban-dictionary-entry-collector

TABLE I: Evaluation of proposals regarding accuracy, sensitivity, and specificity (in%)

	**Naive Bayes**	**Logistic Regression**	**Random Forest**	**Bi-LSTM**	**Bi-GRU**
**Accuracy**	68.49	87.09	89.50	90.12	**93.10**
**Sensitivity**	86.47	88.41	81.64	87.53	**92.08**
**Specificity**	53.60	86.00	**96.00**	92.41	93.96

we expected that the specificity would achieve highest values than the sensitivity.

V. Related work

Scholarly coverage on the specific topic of automatic misogyny detection using deep learning is relatively nascent. Studies either tend focus on hate speech generally and typically the use of more conventional machine learning. With regards to the former, [58] present a study of eight corpora of online hate speech which they analysed for jihadist, extremist, racist, and sexist content. In their study, they test a wide variety of machine learning approaches including deep learning. Notably, they achieved the best results with a deep learning Convolutional Neural Network for sexism detection (c. 95 per cent) but note significant challenges when such systems are used "in the wild" where error rates may not be acceptable.

A paper by Fersini et al. [59] providing an overview of the Evalita18 automatic misogyny identification challenge illustrates the relative use of conventional machine learning versus deep learning techniques. Here, [59] report on a competition where 16 teams competed to identify and classify misogynistic tweets in two languages, English and Italian. The challenge comprised two subtasks: (1) misogyny identification: discrimination of misogynistic content from non-misogynistic content, and (2) misogynistic behaviour and target classification: recognition that the targets are specific users or groups of women together and the identification of the type of misogyny i.e. stereotyping and objectification, dominance, derailing, and sexual harassment and threats of violence. Of the 16 teams, only one team used a deep learning approach, StopPropagHate, in which a simple dense neural network was trained. In this competition, the deep learning approach was marginally less accurate when compared against those using more conventional machine learning i.e. logistic regression and SVM. While [59] concluded that misogyny identification problem was satisfactorily addressed, misogynistic behaviour and target classification remained a challenge. The paper concludes with the suggestion that better performance might be possible with larger and better quality data.

Indeed the Evalita18 and IberEval18 challenges are the primary, albeit limited, source of studies on deep learning for misogyny detection primarily due to the availability of a training data set[5]. In [60], a Bi-LSTM with Conditional Random Fields (CRF) is used for misogyny identification task (text classification) for twitter messages (tweets). The experimental results suggest good performance with 78.9 accuracy on English tweets and 76.8 accuracy on Spanish tweets. Ahluwalia et al. [61] trained an LSTM network to classify a tweet as misogynous or not. Here, [61] report that the deep learning approach was not as accurate as an ensemble of classifiers using logistic regression and tree ensemble models. Again, the limited size of the data set and/or the limited length of tweets was cited as possible explanation for modest performance by the deep learning approaches [61].

[5]https://amiibereval2018.wordpress.com/important-dates/data/

A deep learning approach for identifying aggressive and non-aggressive tweets in the context of misogyny detection, misogynistic behaviour and target classification, is applied in [62]. Here, a deep learning approach incorporating a set of linguistic features in to a convoluted neural network architecture is used. Again, reported results have a low accuracy with linguistic features such as sarcasm and humour causing significant classification problems [62].

No studies of misogynism detection based on Urban Dictionary using machine learning, deep learning or other related techniques, were identified.

VI. Conclusions

This paper outlines the preliminary results of using deep learning for automatic misogyny detection, the first such study using an Urban Dictionary data set. Our results suggest that deep learning techniques can achieve better levels of performance when compared to traditional machine learning techniques.

Our findings present a number of contributions and potential avenues for socio-technical research. Firstly, this study is one of the first studies comparing machine learning and deep learning techniques for automatic misogyny detection. Notwithstanding this, we explore a limited number of machine learning techniques. Extant research on Twitter data sets suggests greater accuracy with hybrid approaches [59], e.g. Ensemble of Classifiers, and further research should be to see whether greater accuracy can be achieved in acceptable deliberation times. Secondly, as a first such study on Urban Dictionary, we make a contribution to the wider literature of online misogynism. We explored data from one platform over several years which due to its nature as dictionary is relatively structured. Additional research, including cross-domain evaluation, is required to understand the generalizability and scalability of the models used. A comparison of deep learning techniques for misogyny detection across multiple social media data sets is worthy of exploration to understand over-fitting and training issues. Thirdly, we make a contribution by providing a training data set for Urban Dictionary, a contribution in itself. This is notable on other grounds too. As the misogynistic terms identified within Urban Dictionary represent a data set of neologisms, they can be used for identifying emerging or cloaked misogynistic terms in other data sets e.g. Twitter. Fourthly, we focus only on automatic misogyny detection. As per [59], [60], further work is required to identify the targets of the misogynistic behaviour and the type of behaviour evidenced in the context examined. Urban Dictionary may not be fully appropriate for the former, as it is a dictionary, but have potential value for the latter. Fifthly, we do not evaluate the intensity of the misogynistic speech which, like sentiment analysis, would have value in this context. Finally, for commercial operationalization, in addition to accuracy and scalability, the issue of interpretability of deep learning results needs to be explored to address legal concerns [58].

Misogynism, online and offline, is a major societal issue with very real psychological and physical consequences for those targeted. Online services and platforms that facilitate the spread of misogynistic speech, unintentionally or otherwise, need to take actions to identify such speech and intervene to curb such behaviour. Deep learning is a potential solution for such detection however it is not without its drawbacks not least the impact of error rates on freedom of speech and mislabelling of innocent users.

List of Acronyms

Bi-LSTM Bidirectional Long Short-Term Memory
CRF Conditional Random Fields
GRU Gated Recurrent Unit
LSTM Long Short-Term Memory
RNN Recurrent Neural Networks
SVM Support Vector Machine

Acknowledgment

This work was partly funded by the World Technology Universities Network and the Irish Institute of Digital Business.

References

[1] A. Brown, "What is hate speech? part 1: The myth of hate," *Law and Philosophy*, vol. 36, no. 4, pp. 419–468, 2017.
[2] L. Richardson-Self, "Woman-hating: On misogyny, sexism, and hate speech," *Hypatia*, vol. 33, no. 2, pp. 256–272, 2018.
[3] A. G. Johnson, *The Blackwell dictionary of sociology: A user's guide to sociological language*. Wiley-Blackwell, 2000.
[4] K. Manne, *Down girl: The logic of misogyny*. Oxford University Press, 2017.
[5] L. Mullany and L. Trickett, "Misogyny hate crime evaluation report," 2018, http://www.nottinghamwomenscentre.com/wp-content/uploads/2018/07/Misogyny-Hate-Crime-Evaluation-Report-June-2018.pdf, Last accessed on 2018-12-12.
[6] Law Commission, "Hate crime," 2018, https://www.lawcom.gov.uk/project/hate-crime/, Last accessed on 2018-12-12.
[7] K. Mantilla, "Gendertrolling: Misogyny adapts to new media," *Feminist Studies*, vol. 39, no. 2, pp. 563–570, 2013.
[8] J. Bartlett, R. Norrie, S. Patel, R. Rumpel, and S. Wibberley, "Misogyny on twitter," *Demos*, 2014.
[9] J. Burgess and A. Matamoros-Fernández, "Mapping sociocultural controversies across digital media platforms: one week of# gamergate on twitter, youtube, and tumblr," *Communication Research and Practice*, vol. 2, no. 1, pp. 79–96, 2016.
[10] M. Mondal, L. A. Silva, and F. Benevenuto, "A measurement study of hate speech in social media," in *Proceedings of the 28th ACM Conference on Hypertext and Social Media*. ACM, 2017, pp. 85–94.
[11] Amnesty International, "Toxic twitter - a toxic place for women," 2018, https://www.amnesty.org/en/latest/research/2018/03/online-violence-against-women-chapter-1/, Last accessed on 2018-12-12.
[12] R. Fulper, G. L. Ciampaglia, E. Ferrara, Y. Ahn, A. Flammini, F. Menczer, B. Lewis, and K. Rowe, "Misogynistic language on twitter and sexual violence," in *Proceedings of the ACM Web Science Workshop on Computational Approaches to Social Modeling (ChASM)*, 2014.
[13] M. Duggan, "Online harassment 2017," 2017, http://www.pewinternet.org/2017/07/11/online-harassment-2017/, Last accessed on 2018-12-12.
[14] Olivia Solon, "Google's bad week: Youtube loses millions as advertising row reaches us," 2017, https://www.theguardian.com/technology/2017/mar/25/google-youtube-advertising-extremist-content-att-verizon, Last accessed on 2018-12-12.
[15] R. Binns, M. Veale, M. Van Kleek, and N. Shadbolt, "Like trainer, like bot? inheritance of bias in algorithmic content moderation," in *International Conference on Social Informatics*. Springer, 2017, pp. 405–415.
[16] K. Crawford and T. Gillespie, "What is a flag for? social media reporting tools and the vocabulary of complaint," *New Media & Society*, vol. 18, no. 3, pp. 410–428, 2016.
[17] Urban Dictionary, "Get wordy," 2018, http://ads.urbandictionary.com/, Last accessed on 2018-12-12.
[18] D. Samuels, "Language, meaning, modernity, and doowop," *SEMIOTICA-LA HAYE THEN BERLIN-*, vol. 149, pp. 297–324, 2004.
[19] J. Patel, S. Shah, P. Thakkar, and K. Kotecha, "Predicting stock and stock price index movement using trend deterministic data preparation and machine learning techniques," *Expert Systems with Applications*, vol. 42, no. 1, pp. 259–268, 2015.
[20] G. Manogaran and D. Lopez, "A survey of big data architectures and machine learning algorithms in healthcare," *International Journal of Biomedical Engineering and Technology*, vol. 25, no. 2-4, pp. 182–211, 2017.
[21] Y. Lu, "Industry 4.0: A survey on technologies, applications and open research issues," *Journal of Industrial Information Integration*, vol. 6, pp. 1–10, 2017.
[22] T. Lynn, P. Rosati, and t. . T. n. . https://www.researchgate.net/publication/328962066_Towards_the_Intelligent_Internet_of_Everything_Observations_on_Multi-disciplinary_Challenges_in_Intelligent_Systems_Research, Last accessed on 2018-12-12. Endo, Patricia Takako year = 2018.
[23] B. Shickel, P. J. Tighe, A. Bihorac, and P. Rashidi, "Deep ehr: A survey of recent advances in deep learning techniques for electronic health record (ehr) analysis," *IEEE journal of biomedical and health informatics*, vol. 22, no. 5, pp. 1589–1604, 2018.
[24] G. L. Santos, P. T. Endo, M. F. F. da Silva Lisboa, L. G. F. da Silva, D. Sadok, J. Kelner, T. Lynn *et al.*, "Analyzing the availability and performance of an e-health system integrated with edge, fog and cloud infrastructures," *Journal of Cloud Computing*, vol. 7, no. 1, p. 16, 2018.
[25] K. Greff, R. K. Srivastava, J. Koutník, B. R. Steunebrink, and J. Schmidhuber, "Lstm: A search space odyssey," *IEEE transactions on neural networks and learning systems*, vol. 28, no. 10, pp. 2222–2232, 2017.
[26] Y. LeCun, Y. Bengio, and G. Hinton, "Deep learning," *nature*, vol. 521, no. 7553, p. 436, 2015.
[27] H. Sak, A. Senior, and F. Beaufays, "Long short-term memory recurrent neural network architectures for large scale acoustic modeling," in *Fifteenth annual conference of the international speech communication association*, 2014.
[28] M. Sundermeyer, R. Schlüter, and H. Ney, "Lstm neural networks for language modeling," in *Thirteenth annual conference of the international speech communication association*, 2012.
[29] S. Xingjian, Z. Chen, H. Wang, D.-Y. Yeung, W.-K. Wong, and W.-c. Woo, "Convolutional lstm network: A machine learning approach for precipitation nowcasting," in *Advances in neural information processing systems*, 2015, pp. 802–810.
[30] J. Heaton, N. G. Polson, and J. H. Witte, "Deep learning in finance," *arXiv preprint arXiv:1602.06561*, 2016.
[31] J. Chung, C. Gulcehre, K. Cho, and Y. Bengio, "Empirical evaluation of gated recurrent neural networks on sequence modeling," *arXiv preprint arXiv:1412.3555*, 2014.
[32] C. Junyoung, C. Gulcehre, K. Cho, and Y. Bengio, "Gated feedback recurrent neural networks," in *Proceedings of the 32Nd International Conference on International Conference on Machine Learning - Volume 37*, ser. ICML'15, 2015, pp. 2067–2075.
[33] R. Zhao, R. Yan, J. Wang, and K. Mao, "Learning to monitor machine health with convolutional bi-directional lstm networks," *Sensors*, vol. 17, no. 2, p. 273, 2017.
[34] I. Goodfellow, Y. Bengio, A. Courville, and Y. Bengio, *Deep learning*. MIT press Cambridge, 2016, vol. 1.
[35] T. Gunasegaran and Y. Cheah, "Evolutionary cross validation," in *2017 8th International Conference on Information Technology (ICIT)*, May 2017, pp. 89–95.
[36] C. Tantithamthavorn, S. McIntosh, A. E. Hassan, and K. Matsumoto, "An empirical comparison of model validation techniques for defect prediction models," *IEEE Trans. Softw. Eng.*, vol. 43, no. 1, pp. 1–18, Jan. 2017.
[37] J. D. Rodriguez, A. Perez, and J. A. Lozano, "Sensitivity analysis of k-fold cross validation in prediction error estimation," *IEEE transactions on pattern analysis and machine intelligence*, vol. 32, no. 3, pp. 569–575, 2010.

[38] Y. Jung, "Multiple predicting k-fold cross-validation for model selection," *Journal of Nonparametric Statistics*, vol. 30, no. 1, pp. 197–215, 2018.

[39] A. McCallum, K. Nigam *et al.*, "A comparison of event models for naive bayes text classification," in *AAAI-98 workshop on learning for text categorization*, vol. 752, no. 1. Citeseer, 1998, pp. 41–48.

[40] S. Chakrabarti, S. Roy, and M. V. Soundalgekar, "Fast and accurate text classification via multiple linear discriminant projections," *The VLDB journal*, vol. 12, no. 2, pp. 170–185, 2003.

[41] D. W. Hosmer Jr, S. Lemeshow, and R. X. Sturdivant, *Applied logistic regression*. John Wiley & Sons, 2013, vol. 398.

[42] A. Genkin, D. D. Lewis, and D. Madigan, "Large-scale bayesian logistic regression for text categorization," *Technometrics*, vol. 49, no. 3, pp. 291–304, 2007.

[43] L. Breiman, "Random forests," *Machine learning*, vol. 45, no. 1, pp. 5–32, 2001.

[44] A. Prinzie and D. Van den Poel, "Random forests for multiclass classification: Random multinomial logit," *Expert systems with Applications*, vol. 34, no. 3, pp. 1721–1732, 2008.

[45] J. Ham, Y. Chen, M. M. Crawford, and J. Ghosh, "Investigation of the random forest framework for classification of hyperspectral data," *IEEE Transactions on Geoscience and Remote Sensing*, vol. 43, no. 3, pp. 492–501, 2005.

[46] E. Frank and R. R. Bouckaert, "Naive bayes for text classification with unbalanced classes," in *European Conference on Principles of Data Mining and Knowledge Discovery*. Springer, 2006, pp. 503–510.

[47] G. Ifrim, G. Bakir, and G. Weikum, "Fast logistic regression for text categorization with variable-length n-grams," in *Proceedings of the 14th ACM SIGKDD international conference on Knowledge discovery and data mining*. ACM, 2008, pp. 354–362.

[48] C. Seger, "An investigation of categorical variable encoding techniques in machine learning: binary versus one-hot and feature hashing," 2018.

[49] Z. Wood-Doughty, N. Andrews, and M. Dredze, "Convolutions are all you need (for classifying character sequences)," in *Proceedings of the 2018 EMNLP Workshop W-NUT: The 4th Workshop on Noisy User-generated Text*, 2018, pp. 208–213.

[50] T. Young, D. Hazarika, S. Poria, and E. Cambria, "Recent trends in deep learning based natural language processing," *ieee Computational intelligenCe magazine*, vol. 13, no. 3, pp. 55–75, 2018.

[51] S. J. Reddi, S. Kale, and S. Kumar, "On the convergence of adam and beyond," 2018.

[52] D. P. Kingma and J. Ba, "Adam: A method for stochastic optimization," *arXiv preprint arXiv:1412.6980*, 2014.

[53] N. Lu, X. Ren, J. Song, and Y. Wu, "Visual guided deep learning scheme for fall detection," in *Automation Science and Engineering (CASE), 2017 13th IEEE Conference on*. IEEE, 2017, pp. 801–806.

[54] K. J. Van Stralen, V. S. Stel, J. B. Reitsma, F. W. Dekker, C. Zoccali, and K. J. Jager, "Diagnostic methods i: sensitivity, specificity, and other measures of accuracy," *Kidney international*, vol. 75, no. 12, pp. 1257–1263, 2009.

[55] K. Wang, G. Cao, D. Meng, W. Chen, and W. Cao, "Automatic fall detection of human in video using combination of features," in *Bioinformatics and Biomedicine (BIBM), 2016 IEEE International Conference on*. IEEE, 2016, pp. 1228–1233.

[56] Y. Fan, M. D. Levine, G. Wen, and S. Qiu, "A deep neural network for real-time detection of falling humans in naturally occurring scenes," *Neurocomputing*, vol. 260, pp. 43–58, 2017.

[57] Y. Fan, G. Wen, D. Li, S. Qiu, and M. D. Levine, "Early event detection based on dynamic images of surveillance videos," *Journal of Visual Communication and Image Representation*, vol. 51, pp. 70–75, 2018.

[58] T. De Smedt, S. Jaki, E. Kotzé, L. Saoud, M. Gwóźdź, G. De Pauw, and W. Daelemans, "Multilingual cross-domain perspectives on online hate speech," *arXiv preprint arXiv:1809.03944*, 2018.

[59] E. Fersini, D. Nozza, and P. Rosso, "Overview of the evalita 2018 task on automatic misogyny identification (ami)," *Proceedings of the 6th evaluation campaign of Natural Language Processing and Speech tools for Italian (EVALITA18), Turin, Italy. CEUR. org*, 2018.

[60] I. Goenaga, A. Atutxa, K. Gojenola, A. Casillas, A. D. de Ilarraza, N. Ezeiza, M. Oronoz, A. Pérez, and O. P. de Vinaspre, "Automatic misogyny identification using neural networks," in *Proceedings of the Third Workshop on Evaluation of Human Language Technologies for Iberian Languages (IberEval 2018), co-located with 34th Conference of the Spanish Society for Natural Language Processing (SEPLN 2018). CEUR Workshop Proceedings. CEUR-WS. org, Seville, Spain*, 2018.

[61] R. Ahluwalia, H. Soni, E. Callow, A. Nascimento, and M. De Cock, "Detecting hate speech against women in english tweets," in *Proceedings of Sixth Evaluation Campaign of Natural Language Processing and Speech Tools for Italian. Final Workshop (EVALITA 2018), Turin, Italy. CEUR. org*, 2018.

[62] S. Frenda, "The role of sarcasm in hate speech. a multilingual perspective."
